# Phospholipase PLA2G16 Accelerates the Host Interferon Signaling Pathway Response to FMDV

**DOI:** 10.3390/v17070883

**Published:** 2025-06-23

**Authors:** Bingjie Sun, Xiaodong Qin, Taoqing Zhang, Sujie Dong, Yinbo Ye, Changying Wang, Yan Zhang, Rongzeng Hao, Yi Ru, Hong Tian, Haixue Zheng

**Affiliations:** State Key Laboratory for Animal Disease Control and Prevention, National Foot and Mouth Disease Reference Laboratory, Lanzhou Veterinary Research Institute, Chinese Academy of Agricultural Sciences, Lanzhou 730046, China; sunbingjie0427@163.com (B.S.);

**Keywords:** PLA2G16, FMDV, STAT1 phosphorylation, innate immunity

## Abstract

PLA2G16 is a member of the phospholipase A2 family that catalyzes the generation of lysophosphatidic acids (LPAs) and free fatty acids (FFAs) from phosphatidic acid. Previously, PLA2G16 was found to be a host factor for picornaviruses. Here, we discovered that the Foot-and-Mouth Disease Virus (FMDV) infection led to an elevation in PLA2G16 transcription. We established PLA2G16 overexpression and knockdown cell lines in PK-15 cells to investigate the potential role of PLA2G16 in FMDV infection. Our findings revealed that during FMDV infection, PLA2G16-overexpressing cells had increased levels of phosphorylated STAT1 and the interferon-stimulating factors ISG15 and ISG56. In PLA2G16-overexpressing cells, p-STAT1 was observed at higher levels and earlier than in wild-type cells. Subsequent research demonstrated that PLA2G16 specifically promoted an antiviral innate immune response against FMDV. The host could detect the early release of FMDV viral nucleic acid in PLA2G16-overexpressing cells and trigger the interferon signaling pathway. Additionally, we discovered that the supernatants of PLA2G16-overexpressing cells stimulated the production of higher levels of ISG56 and phosphorylated STAT1. This suggests that PLA2G16-overexpressing cells can activate the innate immune pathway of uninfected cells after FMDV infection.

## 1. Introduction

Foot-and-Mouth Disease (FMD) is a highly contagious viral disease that affects domestic and wild even-toed ungulates, including cattle, sheep, goats, pigs, deer and African buffalo [1]. High fever and vesicular lesions on the tongue, lips, teats, nose, and feet are the disease’s hallmarks [2,3]. FMDV is a positive-sense single-stranded RNA virus belonging to the Aphthovirus genus within the Picornaviridae family [4].The viral genome consists of a small, non-enveloped nucleic acid approximately 8.3 kb in length, including a long 5′-untranslated region (5′-UTR), a large open reading frame (ORF), and a short 3′UTR [5,6]. FMDV-encoded proteases (Lpro, 2A, and 3C) cleave the polyprotein encoding the ORF into eight non-structural proteins (Lpro, 2A, 2B, 2C, 3A, 3B, 3C, and 3D) and four structural proteins (VP1, VP2, VP3, and VP4) [7]. The structural proteins are essential for viral particle assembly and infectivity, while the non-structural proteins play critical roles in viral replication, polyprotein processing, and virion assembly [8]. Additionally, FMDV comprises seven serotypes: O, A, C, SAT1, SAT2, SAT3, and Asia 1 [9]. Notably, there is limited cross-protection between different serotypes and even between strains within the same serotype.

Interferons (IFNs) mediate innate immune response and provide a strong first line of defense against pathogen invasion [10]. I-IFN and II-IFN signal through different receptors, but both types of IFN initiate a signaling cascade through the Janus kinase (JAK)-STAT pathway, which induces the transcriptional expression of interferon-stimulated genes (ISGs), and the resulting ISGs, along with other molecules produced by the IFN stimulus (pro-inflammatory cytokines, etc.) [11,12]. STAT1 is a member of the STAT family of proteins that mediate I-IFN- and II-IFN-induced antiviral immune responses. Prior to cytokine stimulation, STAT1 is mainly localized in the cytoplasm, and when the receptor is activated by IFNs, the receptor-associated JAK kinase phosphorylates STAT1 to produce p-STAT1, which is commonly used as an indicator molecule for the activation of the interferon signaling pathway [13,14]. The mammalian STAT family has seven members: STAT1, STAT2, STAT3, STAT4, STAT5 (STAT5A and STAT5B) and STAT6 [15,16]. The STAT protein family is a family of transcription factors that regulates signaling transduction and gene transcription, mediating numerous essential aspects of cellular functions such as differentiation, cell death and proliferation [17,18].

Phospholipids are the basic molecules that make up biological membranes [19]. Phospholipases are a class of enzymes responsible for the metabolism of phospholipids in living organisms and are involved in the hydrolysis of various phospholipids, which can be classified into four major groups according to their hydrolysis of phospholipids at different sites: PLA (including A1 and A2), PLB, PLC and PLD [20]. PLA2 is the most frequently detected phospholipase and the PLA2 family has been classified into 16 taxa (taxon I to taxon XVI) based on the time of discovery [21]. The biological functions of phospholipases include maintenance and repair of cell membrane structure, regulation of intracellular metabolic mechanisms and signaling, and digestion of phospholipids in vivo, which have been extensively studied in oncology, metabolism and inflammation [22,23].

Phospholipases hydrolyzes phospholipids to produce a variety of lipid products that regulate many cellular signals and play important roles in physiology, metabolism, and cancer [24]. In recent years, more and more studies have shown that various phospholipases are important participants in the process of viral infection, which is another brand-new direction to study the biological functions of phospholipases. For example, phosphatidylserine-specific phospholipase A1 (PLA1A), a novel host factor for hepatitis C virus (HCV) assembly, is involved in initiating viral assembly in the vicinity of core-modified lipid droplets by binding the HCV replication complex to the envelope complex [25]. PLA2G16 was previously identified as an important host factor for some members of the Picornavirus family [26]. Other phospholipase A2 family members, including PLA2G4A, PLA2G4C, and PLA2GXIIB, participate in HCV replication through multiple different mechanisms [27,28]. PLA2G16, also known as AdPLA, is the rate-found enzyme in the biosynthesis process of free fatty acid (FFA) and is highly expressed, specifically in adipocytes [29]. PLA2G16 has been shown to play a role in prostate cancer, colorectal cancer and pancreatic cancer [30]. In recent years, PLA2G16 was found to be the host factor of picornaviruses [31]. Exploring whether phospholipases have exerted their functions in the invasion process of viral adsorption, replication, assembly, and outgrowth also provides a novel perspective on the infection mechanism of viruses.

In this work, we discovered that the PLA2G16 transcription level was significantly elevated by FMDV infection. In order to explore the potential involvement of PLA2G16 in the FMDV infection process, we constructed PLA2G16 overexpression and knockdown cell lines in PK-15 cells, respectively. During the FMDV infection phase, it was discovered that PLA2G16-overexpressing cells had much higher levels of phosphorylated STAT1 and ISG15 and ISG56 expression than wild-type cells. By triggering the interferon signaling pathway, PLA2G16-overexpressing cells increased the levels and caused an earlier emergence of p-STAT1 in comparison to wild-type cells. Besides FMDV, we used the most widely used model virus, vesicular stomatitis virus (VSV), to infect cells to determine whether the promoting effect of PLA2G16 on the host cell’s innate immune signaling pathway is universal. Subsequent investigations have found that in PLA2G16-overexpressing cells, the FMDV genome is released in advance, which can be detected by the host and leads to the activation of the interferon signaling pathway. Additionally, the cell supernatants of PLA2G16-overexpressing cells infected with FMDV could efficiently trigger the innate immune signaling pathway in uninfected cells.

## 2. Materials and Methods

### 2.1. Cells and Viruses

PK-15 cells and HEK293T cells were purchased from the American Type Culture Collection (ATCC) and cultured in Dulbecco’s modified Eagle’s medium (DMEM, Gibco, Waltham, MA, USA) supplemented with 10% fetal bovine serum (Biological Industries, Beit H, Bioind) and 1% penicillin–streptomycin–gentamicin solution (100×). Modified Eagle’s medium (DMEM, Gibco, Waltham, MA, USA) and the cells were incubated at 37 °C in a humidified 5% CO_2_ incubator (Thermo Fisher Scientific, Waltham, MA, USA). FMDV type O strain O/BY/CHA/2010 was used for the viral challenge. The VSV-G pseudotyped lentiviral particles prepared in our laboratory were used for the viral challenge.

### 2.2. Construction of Plasmids and Stable Cell Lines

The porcine PLA2G16 gene was cloned into the pLV-puro-HA vector, resulting in the construction of the recombinant plasmid pLV-puro-PLA2G16-HA. The primers are shown in Table S1. And the gene was analyzed and verified through DNA sequencing. The plasmids used were extracted using an endotoxin-free plasmid miniprep medium kit (Tiangen, Beijing, China). 293T cells that had undergone fewer than 10 passages were selected for lentivirus packaging when the cell confluence reached approximately 70%. The objective plasmids and packaging plasmids (psPAX2 and pMD2.G) were mixed in Opti-MEM (Gibco, Waltham, MA, USA) at a ratio of 4 µg:1 µg:2 µg. Transfection reagents were subsequently added, and the mixture was incubated for 15 min before being introduced into the cell culture medium. Then, PK-15 cells were infected with lentiviral particles, and positive cells were screened for stable expression with puromycin (InvivoGen, MP, Toulouse, France), and expansion culture was continued after passaging treatment to obtain stable transfected cell lines.

### 2.3. Protein Immunoblotting

Total proteins were extracted with cell lysis buffer (RIPA) supplemented with protease and phosphatase inhibitors (APExBIO, Houston, TX, USA), and the proteins were separated by sodium dodecyl sulfate-polyacrylamide gel electrophoresis (12% SDS-PAGE) and transferred to polyvinylidene difluoride (PVDF) filter membranes, followed by blotting, 1:1000 dilution of primary antibody and overnight incubation at 4 °C. Rabbit-anti-p-STAT1, rabbit-anti-STAT1, and mouse-anti-HA antibodies were purchased from Cell Signaling Technology, Danvers, MA, USA. Mouse-anti-GAPDH were purchased from Abcam, Cambridge, UK. The O-type FMDV VP1 antibody was prepared by our laboratory. The antigen–antibody complexes were detected using an enhanced chemiluminescence (ECL) system (HYK1005, MCE, Monmouth, NJ, USA).

### 2.4. RNA Extraction and RT-qPCR

Total RNA was extracted from the cells using Nucleozol reagent (MNG, Duren, Germany) and the concentration was determined. cDNAs were synthesized using the PrimeScrip RT reagent Kit (Takara, Kyoto, Japan) according to the manufacturer’s instructions. Real-time fluorescence quantitative PCR was performed using THUNDERBIRD^®^ Next SYBR^®^ qPCR Mix (Toyobo, Osaka, Japan) as follows: each cDNA was denatured at 95 °C for 10 s, and amplified for 40 cycles at 95 °C for 10 s and 60 °C for 30 s. The melting curves were 95 °C for 10 s, 60 °C for 1 min, and 95 °C for 15 s. GAPDH was selected as the reference gene because it is constitutively highly expressed and stable in most cell types. Through pre-experiments, it was verified that there was no significant difference in the ct value of GAPDH among the different treatment groups. As the amplification efficiencies of the target gene and the reference gene were 98% and 95%, respectively, the difference was within the acceptable range. The comparative cycle threshold (2^−ΔΔCT^) method was used to calculate the relative expression level [32]. The primers are shown in Table S2.

### 2.5. Interferon Stimulation and GSK8612 Inhibition Assays

IFN-α and IFN-γ were purified and obtained from our laboratory, and wild-type and PLA2G16-overexpressing cells were stimulated with a final concentration of 60 ng/mL and 6 ng/mL, respectively, for 1 h, and then cell samples were collected for the subsequent assay.

GSK8612 (Selleck, Houston, TX, USA) is a TBK1 inhibitor. Wild-type and PLA2G16-overexpressing cells were infected 1 h with FMDV, the culture medium was replaced, and GSK8612 (25 μM) was administered to the experimental group, while DMSO was added to the control group. Cell samples were harvested at 12 h and 16 h after FMDV infection for protein analysis via Western blotting.

### 2.6. Immunofluorescence

PK-15 cells and PLA2G16-overexpressing cells were fixed with 4% paraformaldehyde, permeabilized with 0.02% Triton X-100 and blocked with 10% goat serum. The cells were then incubated with antibodies (1:100 dilution) overnight at 4 °C and stained with AlexaFluor594-conjugated secondary antibodies (1:1000, ab150116, Abcam, Cambridge, UK) for 1 h at room temperature, and the nuclei were stained with 4,6-diamidino-2-phenylindole (DAPI). Images were captured on a confocal laser scanning microscope with a 100× objective (Oxford Andorra, Belfast, Northern Ireland, UK).

### 2.7. Supernatant Collection

Wild-type and PLA2G16-overexpressing cells were infected with 0.05 MOI FMDV, an uninfected control was established, and cell supernatants were collected at 4 h and 8 h after FMDV infection and centrifuged at 3000× *g* for 5 min to remove cellular debris. The supernatants were incubated with PK-15 cells and cell samples were collected for subsequent assays.

### 2.8. Data Analysis

All experiments were performed with at least three independent replicates and statistically analyzed using GraphPad Prism 9.3.0 software. For data with two groups, unpaired Student’s t tests were used under the assumption of normality. Data with more than two groups were analyzed by analysis of variance (ANOVA) under assumption of normality. A *p* value of <0.05 was considered statistically significant.

## 3. Results

### 3.1. FMDV Infection Induces Upregulation of PLA2G16 Expression

**Figure 1:**
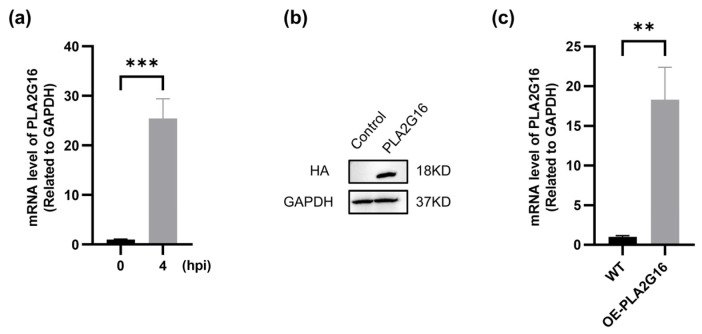
FMDV infection induces upregulation of PLA2G16 expression. (**a**) The PLA2G16 mRNA level of PK-15 cells infected or not infected with FMDV (MOI = 0.05) was detected by RT-qPCR. (**b**) The total proteins of the wild-type and reconstructed cell lines were extracted, and the expression of the HA tag was detected by Western blotting. (**c**) The PLA2G16 mRNA levels of wild-type cells and reconstructed cells were detected by RT-qPCR. The data shown are the mean ± SEM, ** *p* < 0.01, *** *p* < 0.001.

PLA2G16 was previously identified as Coxsackievirus host restriction factor; however, the effect of PLA2G16 on host antiviral signaling pathways was not investigated [31]. According to our research, PLA2G16 transcription was upregulated as a result of FMDV infection (Figure 1a). We created PLA2G16-overexpressing cell lines with HA tags in order to study the function of PLA2G16 in the FMDV infection process (Figure 1b,c).

### 3.2. Overexpression of PLA2G16 Promotes Innate Immune Responses After FMDV Infection

**Figure 2:**
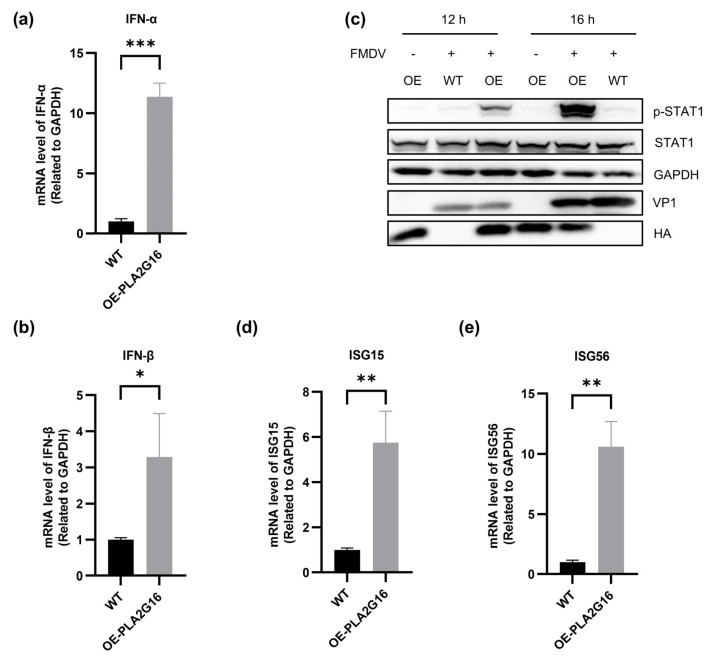
Overexpression of PLA2G16 promotes a natural immune response after FMDV infection. (**a**,**b**) Wild-type cells and PLA2G16-overexpressing cells were assayed for IFN-α and IFN-β mRNA levels after infection with FMDV (MOI = 0.05). (**c**) Cell samples were collected at 12 h and 16 h after FMDV infection (MOI = 0.05) of wild-type cells and PLA2G16-overexpressing cells to detect the level of phosphorylated STAT1 protein. (**d**,**e**) RT-qPCR was performed to detect the difference in mRNA levels of interferon-stimulated genes ISG15 and ISG56 at 16 h after FMDV infection. The data shown are the mean ± SEM; * *p* < 0.05, ** *p* < 0.01, *** *p* < 0.001.

We examined the interferon levels after FMDV infection of wild-type and PLA2G16-overexpressing cells and found that the levels of IFN-α and IFN-β in PLA2G16-overexpressing cells were significantly higher than those in wild-type cells (Figure 2a,b).

The p-STAT1 level and expression levels of ISGs following FMDV infection were then investigated in PLA2G16-overexpressing cells and wild-type cells. The immunoblotting results demonstrated that in PLA2G16-overexpressing cells, FMDV induced significantly higher levels of STAT1 phosphorylation than in wild-type cells (Figure 2c). Subsequently, the transcription of ISGs was analyzed in FMDV-infected wild-type cells and PLA2G16-overexpressing cells. The results showed that, 16 h after FMDV infection, ISG15 and ISG56 were significantly more highly expressed in PLA2G16-overexpressing cells than in wild-type cells (Figure 2d,e).

Thus, we deduce that PLA2G16 overexpression stimulates the host’s innate immune response following FMDV infection because it increases the phosphorylation level of STAT1 as well as the expression levels of ISGs, such as ISG15 and ISG56.

### 3.3. PLA2G16 Promotes FMDV-Induced Phosphorylation STAT1

**Figure 3:**
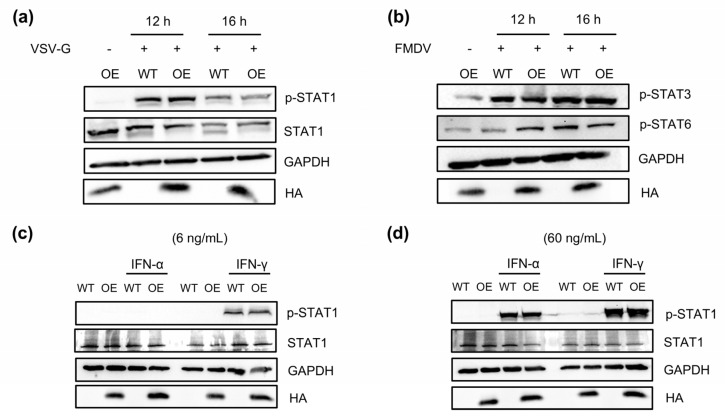
PLA2G16 promotes FMDV-induced STAT1 phosphorylation. (**a**) VSV (MOI = 0.05)-infected wild-type cells and PLA2G16-overexpressing cells; cell samples were collected at 12 h and 16 h after infection, and the level of STAT1 phosphorylation was detected. (**b**) FMDV (MOI = 0.05) was used to infect wild-type cells and PLA2G16-overexpressing cells, and cell samples were collected at 12 h and 16 h after infection to detect STAT3 and STAT6 phosphorylation levels. (**c**,**d**) Wild-type cells and PLA2G16-overexpressing cells were stimulated with IFN-α and IFN-γ at concentrations of 6 ng/mL (**c**), 60 ng/mL for 1 h (**d**).

We also looked at the effect of PLA2G16 overexpression on the VSV in order to determine whether the promoting effect of PLA2G16 on the host cell’s innate immune signaling pathway is universal. We discovered that PLA2G16 overexpression did not promote the upregulation of STAT1 phosphorylation induced by the VSV (Figure 3a). We investigated whether PLA2G16 overexpression might also impact the phosphorylation of other STAT family members; however, the result showed that PLA2G16 overexpression does not promote or inhibit the phosphorylation of STAT3 and STAT6 (Figure 3b).

The addition of IFN-α and IFN-γ to the cell culture medium activates the JAK/STAT1 pathway [33]. To investigate the impact of extracellular interferon on the STAT1 phosphorylation levels, we employed the following approach. Using varying concentrations (6 ng/mL, 60 ng/mL) of IFN-α and IFN-γ, we did not find differences in the level of STAT1 phosphorylation between PLA2G16-overexpressing cells and wild-type cells (Figure 3c,d). We concluded that the promotion effect of PLA2G16 on STAT1 phosphorylation can only be seen with FMDV-infected cells, demonstrating that it does not occur for all RNA viruses.

### 3.4. Overexpression of PLA2G16 Results in Earlier and Higher p-STAT1 Levels

**Figure 4:**
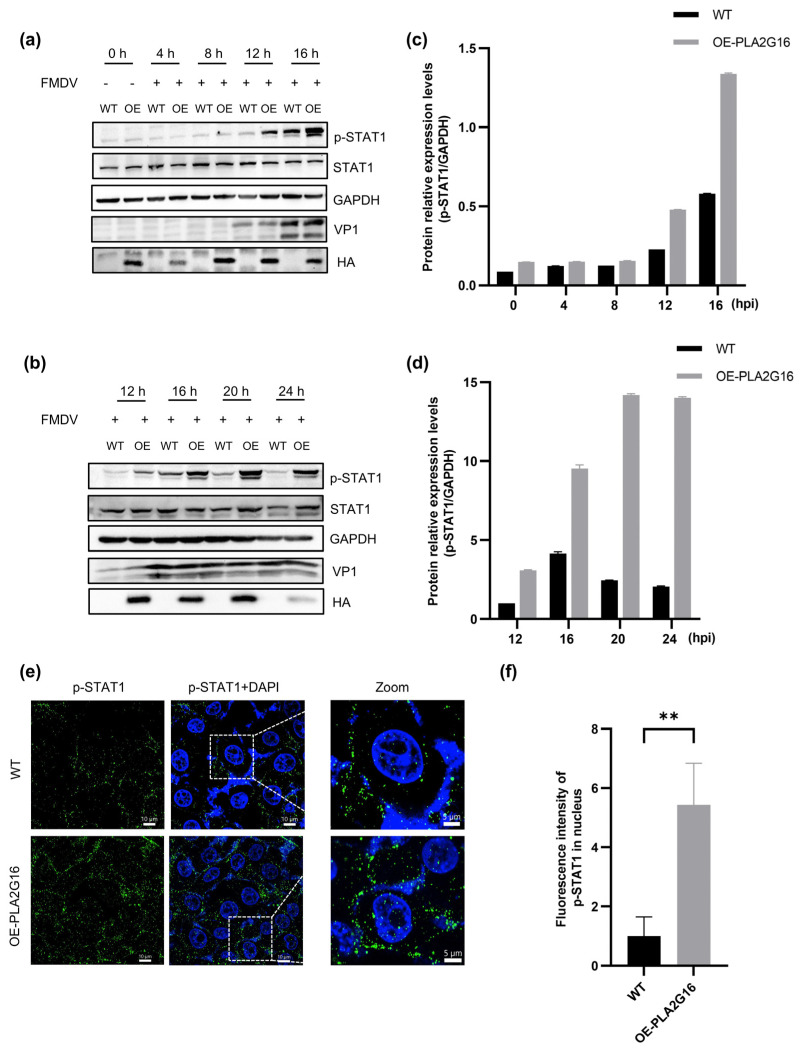
Overexpression of PLA2G16 promotes earlier appearance and higher levels of p-STAT1 after FMDV infection. (**a**,**b**) Cell samples were collected at 0 h, 4 h, 8 h, 12 h, 16 h, 20 h and 24 h after FMDV infection (MOI = 0.05) of wild-type cells and PLA2G16-overexpressing cells to examine the phosphorylation level of STAT1 at different time points. (**c**,**d**) Results of gray-scale analysis of phosphorylated STAT1 protein levels relative to GAPDH at different time points. (**e**) Indirect immunofluorescence was performed after FMDV infection (MOI = 0.05) of wild-type and PLA2G16-overexpressing cells for 4 h, and 4% paraformaldehyde fixation of the cells to detect the expression of fluorescent amount of phosphorylated STAT1 and nucleation. (**f**) Relative quantification of fluorescent expression of p-STAT1 in the nucleus region of wild-type cells and PLA2G16-overexpressing cells. The data shown are the mean ± SEM; ** *p* < 0.01.

At 0 h, 4 h, 8 h, 12 h, 16 h, 20 h, and 24 h post FMDV infection, cell samples from wild-type and PLA2G16-overexpressing cells were harvested in order to precisely examine the alterations in STAT1 phosphorylation. Between 12 h and 24 h after FMDV infection, p-STAT1 was found in both wild-type and PLA2G16-overexpressing cells; the phosphorylated STAT1 levels in the PLA2G16-overexpressing cells were noticeably higher than in the wild-type cells (Figure 4a,b). While phosphorylated STAT1 levels in wild-type cells started to decline as early as 16 h after infection, STAT1 phosphorylation levels in PLA2G16-overexpressing cells continuously increased from 12 h to 24 h following FMDV infection (Figure 4d). PLA2G16 overexpression has no significant effect on FMDV replication, and no difference in the protein level of the structural protein VP1 has been detected. We think this may due to the fact that FMDV has evolved multiple elegant strategies to antagonize host immune response [34].

Immunofluorescence labeling with p-STAT1 antibody also showed that the p-STAT1 signal was much higher in PLA2G16-overexpressing cells than in wild-type cells. Furthermore, we observed that the fluorescence of p-STAT1 in the nucleus of PLA2G16-overexpressing cells was also higher than that of wild-type cells (Figure 4e,f). This is consistent with the immunoblotting results described above, where overexpression of PLA2G16 stimulates FMDV-induced phosphorylation of STAT1. The high fluorescence intensity of p-STAT1 in the nucleus of PLA2G16-overexpressing cells is consistent with the expression levels of ISG56 and ISG15 by RT-qPCR. After phosphorylation by JAK kinases, STAT1 translocated to the nucleus from the cytoplasm as a homodimer and bound to cGAS, and this binding triggered ISGs transcription and expression [35], resulting in high levels of expression of ISG56 and ISG15 in PLA2G16-overexpressing cells.

The above results indicate that overexpression of PLA2G16 contributed to the earlier appearance and higher level of p-STAT1 after FMDV infection, and we speculate that overexpression of PLA2G16 enabled the host to recognize FMDV earlier, thus accelerating the initiation of the innate immune response.

### 3.5. Overexpression of PLA2G16 Results in Earlier Release of Viral Nucleic Acids and Activation of the Interferon Signaling Pathway

**Figure 5:**
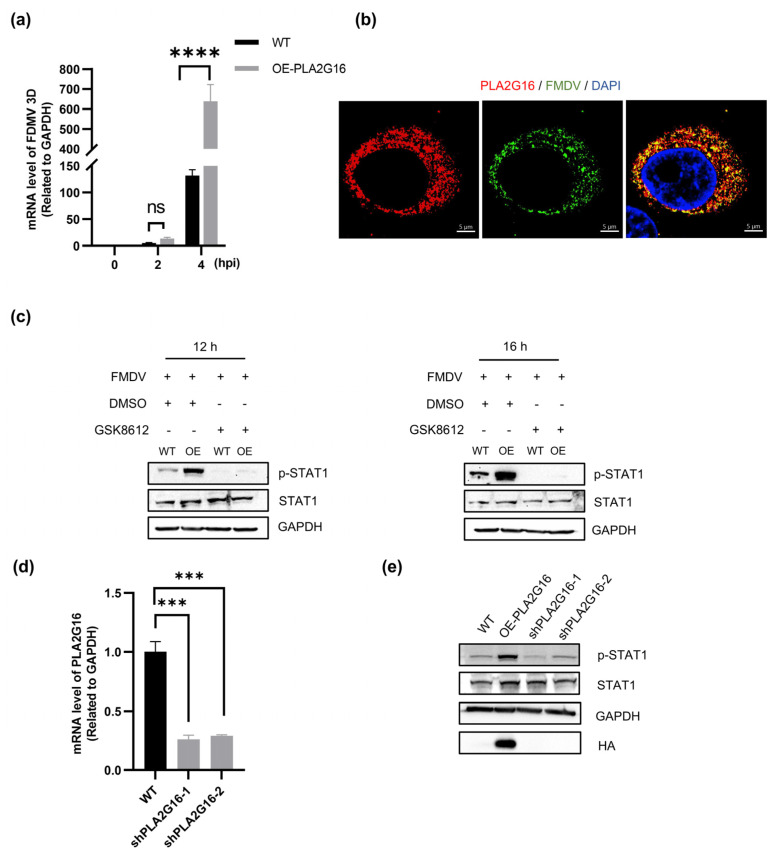
Overexpression of PLA2G16 causes earlier release of viral nucleic acids and the activates interferon signaling pathway. (**a**) FMDV (MOI = 0.05)-infected wild-type cells and PLA2G16-overexpressing cells at 0 h, 2 h and 4 h. Cells were collected for RNA extraction, and RT-qPCR was performed to detect the relative levels of viral nucleic acids. (**b**) PLA2G16-overexpressing cells were examined for co-localization of PLA2G16 with viral particles 4 h after FMDV (MOI = 1) infection. (**c**) Cells were treated with the TBK1 inhibitor GSK8612 (25 μM) or DMSO 1 h after infection with FMDV (MOI = 0.05), and cell samples were collected at 12 h and 16 h post infection to assay phosphorylated STAT1 levels. (**d**,**e**) RT-qPCR assay of PLA2G16 knockdown cells (**d**); 16 h after FMDV (MOI = 0.05) infection of wild-type cells and PLA2G16 knockdown cells, cell samples were collected to detect phosphorylated STAT1 levels (**e**). The data shown are the mean ± SEM; *** *p* < 0.001, **** *p* < 0.0001.

We detected viral nucleic acids at the early stage of FMDV infection and found that the level of viral nucleic acids in PLA2G16-overexpressing cells was significantly higher than that in wild-type cells at 4 h after FMDV infection (Figure 5a). We hypothesize that PLA2G16 overexpression accelerates host detection of viral particles, and promotes the release of viral nucleic acids. According to our hypothesis, early during infection, PLA2G16 will colocalize with viral particles to facilitate the release of viral nucleic acids. Immunofluorescence with FMDV and HA antibodies showed that many viral particles co-localized with PLA2G16 (Figure 5b).

When viral RNA is recognized by pattern recognition receptors, they will activate TANK-binding kinase 1 (TBK1), which is necessary for induction of innate immune response [36]. TBK1 inhibitor GSK8612 treatment blocks STAT1 phosphorylation in both wild-type and PLA2G16-overexpressing cells (Figure 5c) Additionally, we infected the PLA2G16 knockdown cells with FMDV, and the results showed that the level of STAT1 phosphorylation did not rise (Figure 5d,e). In conclusion, PLA2G16-overexpressing cells facilitate the early release of viral nucleic acids, which leads to increased STAT1 phosphorylation, which is dependent on the canonical interferon signaling pathway.

### 3.6. PLA2G16-Overexpressing Cells Infected with FMDV Can Activate Innate Immune Signaling Pathways in Uninfected Cells

**Figure 6:**
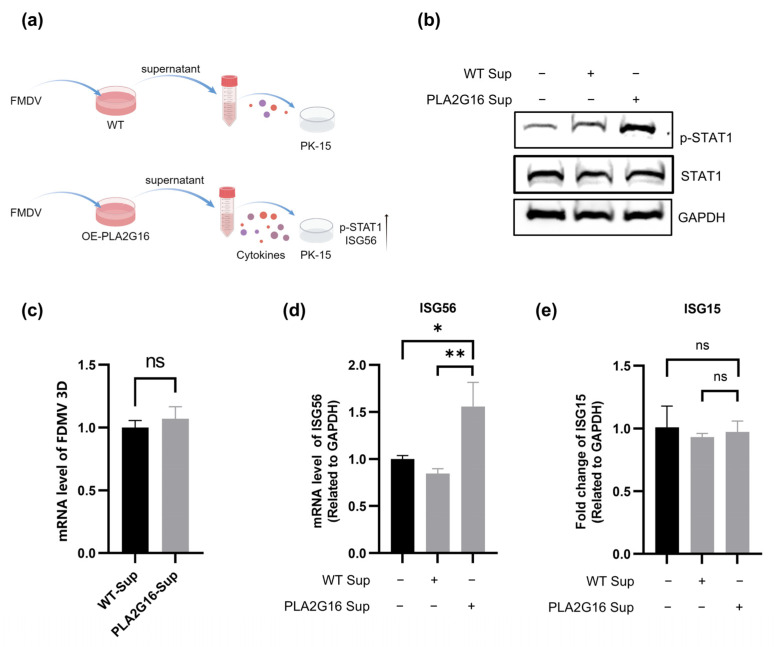
PLA2G16-overexpressing cells infected with FMDV can activate the innate immune pathway of uninfected cells. (**a**) FMDV (MOI = 0.05)-infected wild-type cells and PLA2G16-overexpressing cells at 4 h and 8 h; cell supernatants were collected. The cell supernatants were then incubated with PK-15 cells, and cell samples were collected for subsequent assays; by Figdraw. (**b**) The supernatants of wild-type cells infected with FMDV and PLA2G16-overexpressing cells were incubated with PK-15 cells. Cell samples were collected to extract total protein, and the phosphorylation level of STAT1 was detected by Western blotting. (**c**) The RNA from supernatants of wild-type cells infected with FMDV and PLA2G16-overexpressing cells infected with FMDV; FMDV was then tested by RT-qPCR. (**d**,**e**) The supernatants of wild-type cells infected with FMDV and PLA2G16-overexpressing cells were incubated with PK-15 cells, the total RNA of PK-15 cells was extracted, and the mRNA levels of ISG56 (**d**) and ISG15 (**e**) were detected using RT-qPCR. The data shown are the mean ± SEM; * *p* < 0.05, ** *p* < 0.01.

According to our results, overexpression of PLA2G16 stimulates innate immunological responses following FMDV infection by elevating STAT1 phosphorylation and ISG15 and ISG56 expression levels. Thus, we looked at the biological impact of PLA2G16 overexpression. After infection with FMDV for 4 h and 8 h, cell supernatants of PLA2G16-overexpressing cells and wild-type cells were collected. We incubated the cell supernatants with PK-15 cells. The results showed that the supernatant of PLA2G16-overexpressing cells induce higher level of phosphorylated STAT1 than wild-type cells’ supernatant (Figure 6b). We extracted RNA from the supernatants of wild-type cells infected with FMDV and PLA2G16-overexpressing cells infected with FMDV, and then tested FMDV by RT-qPCR. There was no difference between the supernatants of wild-type cells and PLA2G16-overexpressing cells (Figure 6c). Additionally, the supernatant of the PLA2G16-overexpressing cells stimulates higher ISG56 expression than the supernatant of the wild-type cells, but the supernatant of PLA2G16 had no significant effect on ISG15 (Figure 6d,e). Finally, we conclude that PLA2G16-overexpressing cells infected with FMDV can activate the innate immune pathway of uninfected cells.

## 4. Discussion

Investigating phospholipases’ potential involvement in the invasion process of viral adsorption, replication, assembly, and outgrowth offers a fresh viewpoint on the mechanism of viral infection and represents a whole new avenue for research into the biological role of phospholipases. The phospholipase family comprises a large number of members; however, there are currently few pertinent research reports in the field of virology. As a result, there is a great deal of potential for investigation into the mechanism underlying phospholipases’ function in viral infection. All human enterovirus species studied so far require PLA2G16, which has been discovered to be a host factor for the enterovirus Coxsackievirus; this enzyme is a prospective target for widely acting antiviral medicines [37].

We examined the function of PLA2G16 in the FMDV infection process after discovering in this work that FMDV infection led to a significant increase in PLA2G16 transcription. At 16 h post FMDV infection, we found that PLA2G16-overexpressing cells phosphorylated STAT1 substantially more than wild-type cells in our analysis of important nodes in the innate immune signaling cascade. STAT1 that has been phosphorylated moves into the nucleus and starts the transcriptional production of ISGs. Additionally, we looked at the ISG expression levels and discovered that PLA2G16-overexpressing cells have a much higher level of ISG15 and ISG56 than cells of the wild-type (Figure 2d,e).

In contrast to FMDV, overexpression of PLA2G16 has no obvious effects on VSV induced STAT1 phosphorylation. We induced wild-type and PLA2G16-overexpressing cells with varying IFN-α and IFN-γ doses, and the results demonstrated that there was no difference in the levels of STAT1 phosphorylation (Figure 3c,d). Furthermore, it was not observed that PLA2G16 overexpression increased or decreased STAT3 and STAT6 phosphorylation.

We tested STAT1 phosphorylation at different time points after FMDV infection, and the results showed that p-STAT1 could be detected in both PLA2G16-overexpressing cells and wild-type cells from 12 h to 24 h after infection, and the p-STAT1 level was significantly higher in PLA2G16-overexpressing cells than in wild-type cells (Figure 4a,b). Indirect immunofluorescence results also showed that the intensity of p-STAT1 in PLA2G16-overexpressing cells was significantly higher than that in wild-type cells; p-STAT1 fluorescence intensity in the nucleus was also higher than that in wild-type cells (Figure 4d), and the increased translocation of p-STAT1 into the nucleus initiated the increase in the expression level of ISGs.

We examined viral nucleic acids at an early stage in FMDV infection and found that PLA2G16-overexpressing cells had significantly higher levels of viral RNA than wild-type cells after 4 h of infection, and we also detected co-localization of PLA2G16 with a large number of viral particles (Figure 5a,b). This suggests that overexpression of PLA2G16 promotes the release of viral nucleic acids, thereby contributing to a faster host innate immunity response, but the specific mechanism through which PLA2G16 promotes the release of viral nucleic acids at an early stage is not clear. We speculate that PLA2G16 may induce pore formation in the endosome containing FMDV, increase pore permeability, or enhance pore stability after viral particle entry, thereby accelerating RNA release.

Both nucleic acids and viral proteins of viruses that invade the host can act as triggers for pattern recognition receptors (PRRs). RNA or DNA sensors initiate I-IFN signaling pathways that converge on mitochondrial antiviral signaling proteins (MAVS) and interferon-gene stimulating proteins (STING), respectively [36]. Both MAVS and STING activate TBK1 and inhibit kappa-b kinase (IKKε), which, in turn, activates the transcription factor IFN regulatory factor 3 (IRF3) and regulates I-IFN production [38,39]. We treated wild-type cells and PLA2G16-overexpressing cells with the TBK1 inhibitor GSK8612 after FMDV infection, examined the STAT1 phosphorylation levels at 12 h and 16 h post infection, and found that STAT1 phosphorylation was blocked in both cases (Figure 5c), suggesting that overexpression of PLA2G16 promotes an elevated STAT1 phosphorylation that is dependent on the classical interferon signaling pathway.

We found that overexpression of PLA2G16 activates the classical interferon signaling pathway of host innate immunity. We collected supernatants from wild-type and PLA2G16-overexpressing cells after FMDV infection, and our assay revealed that the expression level of ISG56, along with the phosphorylated STAT1 protein level, was significantly higher after incubation of PLA2G16-overexpressing cell supernatants than that of wild-type cells supernatants (Figure 6b,d). In conclusion, overexpression of PLA2G16 may provide protection against FMDV infection.

## 5. Conclusions

In conclusion, our study showed that overexpression of PLA2G16 enables the host to recognize viral particles more rapidly and promotes the release of viral nucleic acids, allowing the host to mount a more rapid innate immune response. Overexpression of PLA2G16 caused STAT1 phosphorylation to occur earlier and at higher levels by activating with the interferon signaling pathway, and induced the expression levels of ISG15 and ISG56 to be significantly higher than those of wild-type cells. Overexpression of PLA2G16 resulted in faster activation of the innate immune pathway in uninfected cells.

## Data Availability

All data generated or analyzed during this study are included in this published article.

## Supplementary Materials

The following supporting information can be downloaded at: https://www.mdpi.com/article/10.3390/v17070883/s1, Table S1: Primers used in this study for the PCR; Table S2: Primers used in this study for the RT-qPCR.
