# Supplementary material for: Phospholipase PLA2G16 Accelerates the Host Interferon Signaling Pathway Response to FMDV
Source: Viruses. 2025 Jun 23;17(7):883. doi: 10.3390/v17070883 (PMC12299009; doi:10.3390/v17070883)
Supplement: Supplementary file 1 [file viruses-17-00883-s001.zip › viruses-3684996-supplementary.pdf]

Table S1: Primers used in this study for the PCR.

Genes	Sequences (5'-3)
PLA2G16	F：CCGCCCTTTCTACAGACATT<br>R：CGGTGAGTACTTGCCATCAT
shPLA2G16-1	F：GCCATTTACGTTGGCGATGGA<br>R：TCCATCGCCAACGTAAATGGC
shPLA2G16-2	F：GCCATTTACGTTGGCGATGGA<br>R：TCCATCGCCAACGTAAATGGC

Table S2: Primers used in this study for the RT-qPCR.

Genes	Sequences (5'-3)
PLA2G16	F：GAGAACTGCGAGCACTTTGT<br>R：GAGAGCATGACGCCAATGAA
GAPDH	F：ACATGGCCTCCAAGGAGTAAGA<br>R：GATCGAGTTGGGGCTGTGACT
FMDV	F：ACTGGGTTTTACAAACCTGTGA<br>R：GCGAGTCCTGCCACGGA
p-ISG15	F：TCAGAGGTGAGAAGGCTGGT<br>R：GCTTCCTGCAAGTGTCCTTC
p-ISG56	F：CCTGTTGATGGTGCAAAGCT<br>R：TGCACATAGGCTTGAGGTCA
IFN-α	F：CTGCTGCCTGGAATGAGAGCC<br>R：TGACACAGGCTTCCAGGTCCC
IFN-β	F：CACTGGCTGGAATGAAACCG<br>R：AATGGTCATGTCTCCCCTGG
